# The Sharing of Costs and Benefits of Rural Environmental Pollution Governance in China: A Qualitative Analysis through Guanxi Networks Perspective

**DOI:** 10.3390/ijerph19116587

**Published:** 2022-05-28

**Authors:** Yanqiang Du, Pingyang Liu, Shipeng Su, Linyi Zhou

**Affiliations:** 1 College of Public Administration, Nanjing Agricultural University, Nanjing 210095, China; dyqiang2088@njau.edu.cn; 2 Department of Environmental Science and Engineering, Fudan University, Shanghai 200433, China; pyliu@fudan.edu.cn; 3 School of Public Management, Fujian Agriculture and Forestry University, Fuzhou 350002, China; ssp@fafu.edu.cn; 4 School of International and Public Affairs, Shanghai Jiao Tong University, Shanghai 200030, China

**Keywords:** environmental equality, guanxi network, pig farming pollution, cost and benefit sharing, China

## Abstract

Concern has been expressed in many parts of the world that community relations in rural areas are breaking down, making issues such as rural environmental degradation harder to resolve without external regulation. Guanxi is a specific Chinese idiom for characterizing social networks, as a broad term to represent existing relations among people, which can be loosely translated as ‘‘relationship’’. Based on a case study of an underdeveloped mountainous area of Southern China, this paper examined the problem from the perspective of guanxi, and explored the impacts of internal group differentiation catalyzed by pig farming pollution and the subsequent influences on the distribution of costs and benefits of different shareholders. It was found that the guanxi in the village were changed from blood relationship centered to economic interest centered. This disparity exerts a significant influence on the distribution of costs and benefits of pollution control and exacerbates environmental inequalities. This means that pig farmers dominated the narrative of pig farming pollution, while the ordinary villagers chose to suffer without protesting, which hinders the advancement of pollution control, and pig farmers took the benefits of weak pollution control and managed to transfer the external cost to others, while others became direct victims. The paper concludes that the rich become richer and the poor become poorer in both economic and environmental perspectives. It is strongly suggested that guanxi should be integrated into the consideration and decision-making process of rural environmental governance in order to guarantee the efficiency and efficacy of its implementation.

## 1. Introduction

Environmental problems in rural or farming areas have become a major obstacle for creating a “beautiful countryside” and implementing the Rural Revitalization Strategy in China [1,2]. Increasing rural environmental pollution has been shown to threaten the physical and mental health of farmers, as well as regional ecological safety; this poses a serious challenge for state-led rural environmental governance, which has, thus far, failed to address the complex challenge of balancing economic development and environmental pollution control in rural areas [3]. Pig farming pollution is a big challenge for rural environmental protection and sustainable development in rural China. For example, in 2017, 689 million pigs were sold, and the chemical oxygen demand of the livestock and poultry industry reached 100.55 million tons, accounting for 93.8% of the total emissions from agricultural sources. Even though national policies for the control of livestock and poultry pollution were introduced and related public investments increased, many problems remain unsolved, while new problems and conflicts emerge [4,5,6,7].

One easily observable—yet often neglected—factor here is rural guanxi networks. Many scholars note that “guanxi” are extremely important in underdeveloped rural areas of developing countries, particularly when they serve as a mechanism for addressing problems in the transformation of rural society, in the absence of official laws and regulations [8,9]. According to Grootaert’s study on rural Indonesia [10], guanxi networks assist the poor more, because for them, returns on guanxi are higher than they are for the rich. Meanwhile, some scholars find that in rural areas, the rich or the elite have much stronger guanxi networks than the poor, and that the benefits they obtain from them are much greater than those obtained by the poor [11]. In the field of rural environmental governance, studies find that difficulties in implementing rural environmental policies can be attributed to rural guanxi networks, and that these are also closely related to rural social inequities [12]. The traditional guanxi is a very important factor in driving rural environmental improvement in China [13].

Guanxi networks are embedded in every aspect of rural society [9,13,14,15]. As a localized society in nature, rural China’s development and governance mechanisms have been profoundly shaped by guanxi networks [16,17,18]. In the context of rapid urbanization and dramatic rural transformation in China, traditional mechanisms are disintegrating, while market mechanisms have not yet taken shape; therefore, guanxi networks still play an important role [13,19]. Specifically, the influence of guanxi networks on the governance of public rural affairs is highly likely to trigger new social inequalities, thereby further affecting governance efficiency and even social stability. However, these problems have not received adequate attention—practically or theoretically.

Thus, this study attempted to answer the following two issues based on a case study of an underdeveloped mountainous area of Southern China:

(1) how do the guanxi networks among the villagers change by livestock farming?
(2) how do guanxi networks shape the way in which the costs and benefits of pig farming are distributed?

## 2. Literature Review

Guanxi is frequently mentioned in Chinese society, and is also an indispensable analysis perspective [20]. After comparing the social system of China and that of the West, Studies found that Chinese society is neither individual-based nor society-based, but guanxi-based [21]. A large part of the literature suggests that guanxi networks appear particularly important to understand the economy, politics, and culture of Chinese rural society [21,22,23] Literally, it is similar to “relationship”, but with more emphasis on connection and interaction. It is regarded as both “social networks” and “social capital” [16,22], especially in Western literature. In a Chinese context, guanxi also refers to “private contacts”. Studies summarize its fundamental characteristics: (1) as constituted by relations among different actors and may be deemed a series of social contacts of actors; (2) these contacts are of a relatively long duration; and (3) its main function is to offer mutual assistance in need [24].

The controversy surrounding the definition of guanxi remains, and more studies are starting to apply the concept of guanxi inter-disciplinarily because of its strong explanatory power [20,25]. Thus, the purpose of this study was not to provide an accurate understanding of guanxi or relationships, but to use its common understanding with special emphasis on its function and subsequent impacts on rural environmental governance. Based on existing research, our starting point was that guanxi networks comprise interpersonal relationships between individuals and their peers on the basis of ethics, emotions, and interests; it also represents social contacts produced and reproduced in a constant exchange process.

It has been widely discussed that guanxi contributes significantly to rural societies. For example, studies find that guanxi can significantly promote the development of rural enterprises [26]. During the process of urbanization and industrialization, guanxi networks also boost rural labor mobility [27]. In addition, guanxi is critical for the implementation of top-down policies [28] and improves the efficiency and efficacy of rural public goods supply [15].

However, the influence of guanxi networks on rural environmental governance has not attracted much attention [13,29,30]. Unlike relationship networks in Western cultures, guanxi networks in China are not official organizational ones, even though they play a crucial role in rural governance, such as by organizing environmental movements and promoting their success [23]. It has been widely recognized that Chinese top-down environmental governance is faced with serious challenges in rural areas. For example, environmental governance may lead to conflicts between local demands and those prioritized by the central government [31,32]. Furthermore, there are other factors that weaken the government’s policy enforcement capability in rural areas, such as the dispersed, non-point sources of environmental pollution, difficulties in identifying and monitoring pollution sources, the high costs of information access, and the excessive number of actors involved [33,34]. Consequently, more attention should be paid to the rural community itself. Hodge (2001) recommended that agricultural environmental policy should establish a broader institutional framework between different organizations and individuals, including considering rural informal institutions [35]. North (1990) demonstrated that guanxi networks exist as a form of informal rural institution [36]. Therefore, it appears particularly important to explore environmental governance within rural communities from the perspective of guanxi networks [37,38]. Considering the cultural “fertile soil” for the development of guanxi in rural China, it is particularly important to understand the economy, politics, and culture of Chinese rural society from the perspective of guanxi [39].

Rural environmental management is a typical regional public good, and many studies center on its efficiency and efficacy [2,40,41]. Yet, existing studies have not conducted in-depth analyses on the distribution of cost and benefit related to rural environmental treatment among different stakeholders, even though some might be beneficiaries, while others are victims [42]. Hanson argued that an overall high efficiency does not guarantee equality [43]; instead, in the overwhelming majority of cases, unbalanced distribution results in environmental inequalities [44]. For example, considering products/services that are harmful to the environment, the poor often take greater environmental risks to achieve cost-effectiveness, and are unable to enjoy the benefits of environment-friendly products/services [45]. This indicates that the costs of environmental policies disproportionately fall upon low-income groups, while the benefits of environmental policies are often largely reaped by high-income groups [46].

At present, Chinese rural society has become seriously stratified, meaning that the disparity between villagers in income, occupation, and social capital, among other factors, has brought about unequal access to resources, services, and social status. Moreover, it differentially affects villagers’ benefits from the supply of rural public goods (i.e., roads and environmental governance) [31]. It is therefore imperative to systematically investigate the influence of guanxi networks among different groups in Chinese rural society and its effects on the costs and benefits of industry growth and pollution from pig farming, and their distribution.

While not related specifically to China, solving the rural environmental pollution through various ways in many developing countries, such as top-down ways and bottom-up ways, is important. This paper offers a new perspective for better understanding the interaction of guanxi networks and rural environmental governance in China. This may help others to better understand why policy fails in rural pollution prevention. A lack of understanding of guanxi results in an ineffective control of animal waste and the failure of environmental regulations. Instead, improving trust-based networks is a better option to control pig farming pollution and promote environmental justice in China, as well as in similar societies in other countries.

## 3. Materials and Methods

### 3.1. Study Area

The target village for our case study was S village, located in the undeveloped mountainous area in northern Fujian Province, China. The village comprises 248 households, which are divided into 6 villager groups, with a total of 998 villagers. In 2015, its per capita annual income was around 5900 RMB. In 2020, S village had 890 villagers, and its per capita annual income was around 8100 RMB. The village has hardly any collective fiscal revenue, and the situation was exacerbated by the rapid urbanization process. The large scale of population hollowing out led to the collapse of this traditional community, significantly weakening the influence and appeal of the village collective.

In the early 1990s, the pig farming industry was first introduced to S village, and developed rapidly. At the time, many rural migrant workers, who had been working in cities, returned to farm pigs in the village area because of its relatively high profits. Later, the village saw the constant expansion of the scale of pig farming; however, no pollution treatment facilities were built, and pig excrement and urine were discharged directly into local rivers. The local government adopted a series of measures to control this pollution, for example, ordering unqualified households to demolish their farms and subsidizing other households to construct biogas digesters. However, these measures did not work well; on the contrary, as soon as the restrictive government policies were relaxed, the industry rebounded. Many demolished pigpens reappeared, becoming larger and more numerous, causing increasingly serious environmental pollution. The nearby river was sometimes reported to be black and smelly. The village’s drinking water sources were contaminated by waste water several times (subsequently, villagers used mountain spring water instead), which adversely affected the health of villagers.

### 3.2. Data Collection

The data in this study were collected mainly through long-term observations at the village, in-depth interviews, and questionnaire surveys. First, since 2013, a research team of at least four researchers stayed in the village for about two weeks every quarter to observe it. By living together with the villagers, we were able to understand the production, lifestyles, and interpersonal relationships within the community. While observing the principles of understanding and recognizing our respondents’ rights to confidentiality and privacy, we generated data in the form of photos, videos, and village field survey reports, among other data.

Second, a questionnaire survey involving the entire village was conducted from March 2013 to July 2014. All the households (except those unavailable at that time, such as migration workers) were visited and questionnaires were completed face to face, considering the literacy issues among villagers. A total of 110 questionnaires were collected, of which 64 were from farmers (refers to ordinary villagers who do not have pigs), and 46 from pig farmers.

Third, based on the information gained from observation and the questionnaire survey, semi-structured interviews were carried out with key figures in the village using a snowball sampling method. In S village, the main stakeholders included pig farmers, farmers, village committee cadres, and officials of the township government. We conducted 55 interviews, including the village cadres and reputable clan figures. All the interviews were transcribed when finished and translated into English. Deep reading of transcripts was applied for coding and analyzing.

Finally, data from official reports and supplementary interviews by telephone calls were also analyzed. To clarify any ambiguities and double check quantitative data, the lead researcher called the village cadres, reputable clan figures, and some villagers on occasion, from April to December 2016, January 2019, and July 2021.

Based on all the data above, we conducted qualitative research based on a case study. This paper illustrates the role of guanxi in mobilizing resources in rural environmental struggles in China following the bidirectional mechanism framework of guanxi—socioeconomic impacts–environmental behaviors. The extent to which village-level guanxi networks have survived the increasingly narrow market-focused interests of specific villager groups, such as the differentiation between pig farmers and farmers, was analyzed and the subsequent economic, environmental, and social costs and benefits of pig farming were estimated. Finally, the environmental behavior and governance of the villagers were analyzed. While qualitative methods were used, they were mainly used to show the functions of guanxi networks.

## 4. Results

### 4.1. Changing of Guanxi Networks and the Differentiation of Households

#### 4.1.1. The Changing of Guanxi Networks

S village used to be clan-dominated, with characteristics typical of traditional communities. Clan relations played significant roles in traditional rural society to provide many common goods, like roads and education, especially in the Fujian province where clan relations were strong. For example, when a family in difficulty seeks help, it mainly resorts to neighbors, relatives, and friends. Public commodities at a village level, such as the construction of bridges and roads, were also supplied mainly through collective action, which is described by the Chinese saying “those with money contribute money and those with strength give strength.” Addressing changes in rural society, the director of the Women’s Federation in this village said:

*Nowadays, community relations and village regulations are generally unimportant, because people are just judged on their fortune rather than their moral character. (Female, 46, village director of the Women’s Federation)*

However, traditional community governance has been seriously challenged by rapid urbanization and industrialization since the 1980s. As an undeveloped mountainous village, S village used to depend heavily on rice planting, a labor-intensive but low-output crop. The population hollowing out continued among the younger generation and able-bodied men, and households became atomized, driven by maximization of private interests, which led to the collapse of the community. Due to its rapid growth, the pig farming industry became the pillar industry and a major income source for many farmers; this turned out to reshape the community with its economic power. Driven by profits, people joined the industry eagerly. However, not every household had the same opportunities. According to the data from our interviews, there are underlying requirements for pig farming: economically, a large initial capital should be available for investment in building pigsties and buying piglets; socially, information sources on markets, technology, and the availability of types of services are necessary. For farmers with no industry experience and who depend heavily on the community, these prerequisites are beyond their capabilities; therefore, they have to seek help from their guanxi network. In fact, the growth of pig farming in S village can be attributed to relatives or friends “passing on experience, helping, and guiding newcomers.” As farmer (non-pig farmer) C stated in the survey,

*Pig farming in the village depends completely on relatives or friends... because people who have good relationships often discuss the situation with the price of pigs, farming techniques and government policies together. The main reason why most ordinary agricultural households do not raise pigs is that they do not have start-up funds or people to give them technical guidance...These things require help from nearby pig farmers. (Male, 52, farmer)*

Catalyzed by the pig farming industry, the traditional blood relation-based guanxi was gradually replaced by economic interest-centered guanxi. As a result, interpersonal contacts, social morality, and the supply of village-level public commodities were changed significantly. Villagers were always busy making money rather than bonding with each other. When they were short of something, such as labor, they did not seek help from their neighbors or relatives, but rather paid to hire workers. As the compiler of the clan pedigree in the village put it:

*“Over these years, people have just done their own thing…They just maintain so-so relationships with other villagers in this village, and only greet each other, and their relationships with each other are not so close as before. For example, if a family happened to be eating and another person came by, then the host would invite the visitor to dine and drink together. But now the visitor will refuse to dine even if the host adds a pair of chopsticks.” (Male, 56, compiler of the clan pedigree)*

We further discovered that the main reason why villagers are unwilling to eat in other people’s homes is because they are afraid of owing favors (i.e., “I will invite you next time”). This reflects a more pecuniary interest-based relationship, and almost everyone just associates with people they get along with. There are many kinds of guanxi networks in rural China caused by different factors, and this research focused on the guanxi network caused by pig breeding.

#### 4.1.2. The Characteristics of Guanxi Networks in Different Groups

The village was divided by two guanxi groups: pig farmers and farmers. As our survey shows, in S village, there are 46 pig farmers, including 22 large-scale ones, 22 scattered (“san yang”) households, and four households that gave up raising pigs by demolishing their pig farms in July 2014, as they wanted to move to the city. With high incomes, these groups are better able to attract capable people, including young people, to join them. The average age of the pig farming group is 48 years old, and they are mostly men (74% of pig farmers are men). Farmers, who were “excluded” from the sector in the village are the group that do not raise pigs. The traditional industry of S village was crop production, which was highly labor intensive in this mountainous area and brought in little income. Our survey indicated that farmers are 51 years old on average. Our findings indicated that pig farmers are younger, which means that the pig farming industry has a certain attractiveness (Table 1).

Meanwhile, different groups have different kinds of guanxi networks. Pig farmers have stronger guanxi networks and are more capable than farmers who access external resources. As Table 2 indicates, each pig farmer has an average of 1.9 relatives, who are similarly engaged in the industry and earning an average annual household income of 76,349 RMB. The survey indicated that the villager cadres in S village are hardly separable from the pig farming group (17% of pig farmers are or were village cadres). Moreover, all the village cadres in S village are pig farmers, and as “capable people” in the village, pig farmers who successfully make money are more likely to be elected as village cadres. The village cadres were therefore categorized in the “pig farmers” group. In contrast, the number of relatives of farmers is 1.6, and their average annual household income is 54,840 RMB. There is a positive correlation between the scales of the resources. As the pig farming sector has grown, the difference in the return for crop production and animal husbandry has further widened the economic gap between pig farmers and non-farmers.

We also found that pig farming shaped the nature of guanxi in different groups. For the pig farmers, the guanxi network is growing, driven by the economic benefits—this means that they are self-enforcing, being perpetrators; the farmers’ guanxi networks are shrinking, turning them into victims. Through the industry chain, pig farmers are more able to extend their relationships vertically and associate with people outside their family. As a result, they were able to acquire more information and take advantage of resources. Farmers mostly engage in traditional agriculture and associate with a limited group of older people on the basis of blood and geographical relationships; this constrains their access to information and resources. Our interview material indicated this clearly:

*The scope of our associations is limited to our village or some relatives and friends...But pig farmers have much greater interpersonal relationships than us. We often see them inviting their friends to dine and drink at home. Some large pig farming households, in particular, have extremely good relationships with the town government, village cadres, pig dealers and so on. (Male, 49, farmer)*

These different guanxi networks have an impact on the effectiveness of pollution control in the following ways.

(1) Pig farmers gradually expand the scope of their external associations and take advantage of their powerful guanxi to get close to government officials. Through these strong guanxi networks, they are able to obtain information and resources relating to governance policies—for example, biogas digester subsidies or the planned scope of demolition—or the timing of inspections. Some government officials can use their power over pollution control to make a profit. In this way, environmental governance is transformed into a power-for-money deal, and for this reason many top-down pollution control measures are not really implemented. For example, in an interview, a pig farmer indicated:

*Whenever the Spring Festival and other festivals come, I always give some cigarettes and wine to village cadres, and some precious tea to the town leader in charge of this village. My son is the driver for a leader of this town. So other pig farmers also seek connections with my son. (Male, 50, pig farmer)*

(2) Some pig farmers work with each other to resist pollution control. Given their shared economic interests and close relationships within the group, pig farmers have a greater incentive to seek the maximization of group interests. For example, during surprise inspections launched by the government, these households are often informed in advance, and some backyard pig farmers drive their pigs to the pigpens of farmers with larger facilities that they have good relationships with, to thereby bypass the requirements to construct pollution control facilities or biogas digesters.

(3) Farmers have weak guanxi networks and are gradually marginalized in terms of rural public affairs. They are not able to raise pigs themselves or to escape the impact of the pollution caused by other villagers, who do. Facing the strong pig farming group, they can only choose to “keep silent” for the following reasons. First, they must have at least some social contact with pig farmers; they prefer to become a “silent majority”, rather than cause offense, so as to maintain these relationships and put a good face on things. Second, because they are still based on kinship relations, the guanxi networks of farmers are looser and they are marginalized in terms of their ability to speak out on village affairs, especially in contrast to pig farmers. Third, pig farmers have a lot economic power, and farmers must often rely on them for job opportunities or to borrow money, or in other ways. For example, pig farmers may give the dissatisfied victims of pollution some pork, or pay them to work on their farms. As this village is not rich, most farmers who do not raise pigs have accepted pollution, instead of choosing to resist it, as long as they can get some benefits from their pig farming neighbors.

It follows that these different guanxi networks place various groups in the position of being “perpetrators” or “victims”, in terms of their relationship to pollution control. Although pig farmers are the perpetrators of pollution, they can rely on the benefits from pig farming to further strengthen their guanxi networks; farmers, however, end up as “victims” of environmental pollution. Meanwhile, government-led top-down policies persist, even though they are not really implemented, due to the powerful influence of the pig farmers. The vast majority of pig farmers have not even constructed the most basic facilities (such as biogas digesters), and the disadvantaged position and silence of farmers that are excluded from the sector means that government policy is not supported from below.

### 4.2. Influence of Different Guanxi Networks on the Distribution of Costs and Benefits of Pollution Control

The “perpetrator–victim” situation described in the preceding section is just an external characterization of the unsatisfactory effects of pollution control. The different guanxi networks among these two groups also create a situation of “prosperity and poverty”, mainly through their influence on the way that the costs and benefits of pollution control are shared. Although all the members of S village are victims of pig farming pollution, some groups are more capable of evading the consequences of environmental pollution and environmental risks, while others bear a disproportionate share of the costs.

#### 4.2.1. Costs and Benefits to Pig Farmers

The benefits gained by pig farmers come mainly from selling pigs. Although the labor opportunity cost of raising pigs is low, the returns are quite high; this is why many villagers are willing to raise pigs instead of leaving the village for work. We performed telephonic follow-up interviews with some villagers in January 2019 and July 2021, and analyzed the returns on pig farming for 2015 and 2016; the results are shown in Table 3. In terms of pig cost, the gross cost per head of pig on average was 1300 RMB (feed cost 1100 RMB, disease prevention 150 RMB, water and electricity costs 20 RMB, and loss due to pigs that died of disease 30 RMB), the labor input and environmental cost was not calculated. We found that every pig brought a profit of 950 RMB in peak season. Moreover, small-scale farmers could make a net annual profit of 30,000 RMB, and large-scale farmers a net annual profit of 50,000 RMB.

These economic benefits are realized on the basis that environmental costs and social costs are not considered, even though these are considerable. Pig farmers in S village mostly have sties with cement floors, on which excrement and urine accumulate, causing a strong odor. Pig farmers wash the excrement and urine away with clean water twice a day, usually at 8:00–9:00 a.m. and 4:00–5:00 p.m. The waste water is discharged directly into nearby rivers. In this way, they gain an economic advantage by exploiting the ecological environment of the village without paying any cost. The wealth of this group ultimately originates from the loss of natural and social resources.

When faced with breeding pollution control, pig farmers’ income was unchanged, but they experienced a substantial increase in the cost. If governance is required in terms of discharge standards, the pig farmers must bear the costs of aquaculture waste water treatment. For example, it costs approximately 10,000–20,000 RMB to build a biogas digester (the government gives a subsidy of 1200 RMB for every biogas digester), and this is equivalent to the profit that can be made from 40–80 heads of pigs. Because of the lack of supervision and penalties, as well as the mismatch between the costs and benefits, most pig farmers do not construct biogas digesters, but choose to directly—and illegally—discharge waste water into rivers and streams.

#### 4.2.2. Costs and Benefits of Farmers

Farmers bear the brunt of the costs of pollution related to pig farming. The direct losses include a decrease in agricultural production due to pollution, and the pollution of water sources used for drinking and household activities, which means higher costs to buy water. The indirect losses include a drop in the value of their property, as well as the negative social impact. Pollution from pig farming has made S village virtually uninhabitable, and the disappearance of development opportunities has led to a sharp decline in the value of real estate. Moreover, the worsening relationships and conflicts over pig farming are also an invisible burden on villagers, as we discuss further below.

In the early years of the development of pig farming, farmers derived some benefits from it; for example, they could acquire pig manure and urine to use as fertilizer. However, after the scale of the industry expanded, only few farmers got the chance to work as wage laborers on large-scale farms, which paid approximately 1500–2000 RMB/month, while most farmers suffered various direct and indirect losses. Because most pig farmers in S village “save” the cost of treating waste water and directly discharge nearly all pig excrement and urine into rivers, the bodies of water approximate to pig farms have suffered severe eutrophication. The water is not fit for drinking and cannot even be used to irrigate farmland. In our survey, many farmers reported that “our crops die from excessive pig farming waste water”, “rice seedlings grow well but bear no fruit”, and other problems. One farmer complained that crop losses were a heavy burden, because he did not have any other source of income:

*My crops are affected by waste sludge and water from nearby pig farming households, and my rice is all damaged by the excessive phosphorus and nitrogen content in the irrigation water, which is over the standard. I got no harvest this year! (Male, 48, farmer)*

Water sources for household use are also polluted, and water costs are higher. Waste water from pig farming not only affects irrigation water, it also contaminates water in deep wells and rivers through surface and underground penetration. S village therefore has to draw on drinking water from sources higher up the mountain, which has significantly increased the cost of the water supply. The use of polluted surface water and underground water by people or animals is still very difficult to avoid, which might cause potential health problems. A female farmer we interviewed said:

*Nearly all the wells, from which we used to fetch water, could no longer be used. Our current drinking water comes from a mountain (where there are no farms) very far from the village. We laid pipes from that mountain to our homes. The costs were equally shared by our villagers, including pig farmers and other farmers. (Female, 42, farmer* *)*

Farmers’ indirect losses are mainly of three types: (1) Property loss. Pollution from pig farming has made S village almost uninhabitable. Heavy pollution accelerated the hollowing out of the rural population, especially those with knowledge and wealth. Even the pig farmers themselves have moved to cities with the money they earned, leaving hired workers in the village. Although the market value of rural housing is not available as these transactions are forbidden according to China’s laws, it usually takes more than 100 thousand RMB to build a modest house in the village; however, it is highly possible that houses in S village have become negative assets due to the serious pollution. (2) Collapse of community. The irregular development of the pig farming sector has fostered a money-oriented attitude in S village, undermining traditional clan rules and community virtues. There have been many conflicts over pig farming in the community and relationships among different groups have deteriorated. For example, the pig farm of resident R is located near the home of farmer T. T has been complaining to R about the pollution for a long time.

*“You are too unconscionable. Your conscience has been eaten by dogs. If your farm had been there for a year or two, I would not complain to you. But you have been raising pigs for so long here and your farm has always been making that smell. How are we supposed to live here? Do you want me to die in your pigpen?”*

Pig farmer R countered farmer T by saying:

*“This plot of land is mine. I constructed the farm on my own land. If you are dissatisfied with the farm, will you give me another plot of land to build a farm? Moreover, it’s hard for us to make money, like a gamble! You complain this way every day. So no wonder that our pigs do not grow well. You envy my good luck. If you have the ability, you can raise pigs. I would let you to build a similar farm next to our home!”*

(3) Loss of development opportunities. As General Secretary Xi Jinping of China puts it, “Clear waters and lush mountains are invaluable assets.” When serious pollution from pig farming destroys the ecological environment, villagers are deprived of their future, and later generations of the right to development. Unfortunately, however, short-term interests still play the leading role in decision-making in the consciousness of most villagers.

#### 4.2.3. Influence of Guanxi Networks on the Cost and Benefit Sharing of Different Groups

As can been seen in the above analysis, pig farmers do not bear the costs of environmental governance caused by pollution, but often cause innocent farmers to bear more of these costs. The crux of the matter lies in that the pig farmers and farmers are very different in terms of the guanxi networks they can draw on to pursue their interests. As a result, the rich have become richer and the poor have become poorer, and for village S, inequalities in rural environmental governance have worsened.

Specifically, pig farmers transfer the costs of pollution by taking advantage of their relatively strong guanxi networks; in this way, they minimize their direct losses from pollution. If the waste water was to be discharged according to the official standard, they would have to pay for the treatment costs, and their net profits would be greatly reduced. In the case of a sudden decrease in pork prices, they might suffer a loss. Pig farmers, therefore, do everything they can to avoid bearing increased environmental costs (for example, giving township government officials gifts, and performing small favors for the victim group at the Spring Festival and other festivals), and even ingeniously transfer the costs of pollution control to the public. These actions have led to serious negative externalities. Moreover, pig farmers also avoid indirect losses, such as exposure to environmental pollution, by migrating elsewhere in the region. In S village, large-scale pig farmers often damage the environment more seriously, but they have more economic strength, and therefore greater ability to cope with supervision. They have not engaged in raising pigs themselves for a long time and they no longer live in the village. For example, in recent years, some pig farmers have purchased housing in cities in the district or prefecture that have a better environment, thereby avoiding the impact of pollution on their daily lives; this behavior has contributed to a rapid rise in house prices in the local prefecture and district. Pig farmers are therefore not exposed to the negative effects of pollution on their health.

Farmers bear more pollution costs because of their weak guanxi networks. On the one hand, this discourages them to protest or fight back, as they feel nobody would listen to them or support them. They take it for granted that those rich pig farmers have the power to monopolize the right to speak and suppress others. Further, farmers sometimes seek help from pig farmers, such as borrowing money for emergencies or receiving occasional petty favors; these victims can be seen as the “silent majority”. On the other hand, due to their poor financial conditions and weak guanxi network, farmers are rarely able to move to cities to avoid the environmental risks. Thus, they have to stay in the village and bear the costs of environmental governance, hoping that ecological remediation may eventually be possible.

In conclusion, different groups in S village share the costs and benefits of pollution related to pig farming in very unequal ways, and this result is mainly due to different groups’ guanxi networks. The wealth of pig farmers ultimately originates from the conversion of natural and social resources. Compared with farmers, pig farmers have stronger guanxi networks, receive more environmental benefits, and generate greater environmental pressure. In the face of pollution control, pig farmers transfer the costs of pollution by taking advantage of their relatively strong guanxi networks, who have mastered the community’s right to control the relationship between individuals. These strong guanxi networks serve the maximum demand of the interests of pig farmers, but play a negative role in rural environmental governance. A study by Du et al. [2,10] agreed that the guanxi network is the third resource allocation method in rural China, in addition to government and market allocation.

Under the influence of this warped guanxi network, rural environmental governance suffers a double loss: the environment is not governed, and the economic growth is not sustainable. For pig farmers and farmers, both have suffered losses: the environment continues to deteriorate, leading to stricter government supervision and governance measures; finally, more stringent cultured control measures will be adopted to shut down pig farms. In March 2017, the pig farms were forcibly demolished by the local government. The case of rural environmental governance in S village, our results show that the guanxi network-led bottom-up governance and government-led top-down governance still need to find a valid combination path to further promote efficient and sustainable rural environmental governance.

## 5. Discussion

Previous studies found that Guanxi has a significant influence on environmental governance, but the conclusions are different [47]. Some researchers believe that high level social capital have a significant and negative impact on environmental pollution [48,49,50], while other studies draw opposite conclusions [13,51,52]. Specifically, the social capital plays a role through the impact on environmental behavior [53]. In the present study, we found that pig breeding enhanced the guanxi network of pig farmers, which directly led to the gradual deterioration of rural environmental pollution. This finding supports the conclusion that guanxi is not conducive to promote rural environmental protection. The polluter-pays principle is difficult to implement in rural pig breeding China because of pig farmer guanxi networks [54].

Different guanxi networks affect the way in which the costs and benefits of pollution control are shared. As a result, the rich have become richer and the poor have become poorer, and environmental inequalities have worsened [55,56]. The relevant literature showed that stronger social relation networks may lead to stronger willingness to protect the environment [57]. However, it has actually a precondition that the social norms are strong [53,58,59]. Because of the lack of social norms, pig farmers accrue economic advantage by massively transferring environmental costs and become “wealthy perpetrators” while farmers that are forced to bear serious pollution and costs becoming “poor victims”.

This study reflects on the nature and strength of the dyadic personal relationships and networks that are at the heart of Chinese rural culture, and the extent to which they could offer a new way of thinking about rural policy more generally. As this suggests, the role of guanxi and other informal institutions must not be ignored in rural environmental governance. In the changes and differentiation of guanxi in the current rural areas, we should pay more attention on local characteristics and even adopt uniform, institutionalized measures to address the rural environment, such as environmental regulation and ecological remediation policies, which have been proposed to address these issues. Besides, guanxi networks lead to costs and benefits being shared among different groups, which, in turn, leads to environmental inequities. Farmers, as the environmentally vulnerable groups, should be should be targeted by government policies.

In concluding, therefore, this paper offers new insights to an extant literature on global rural change. At its core, the issue is that not only is traditional class habitus and place identity declining in rural areas, but that it is being replaced by new, plural forms of network and association that challenge not only local cultures, but also the institutions that have provided stable governance. This is a significant finding given the governance of pollution from pig farming and others, in China and elsewhere, that often drive rural environmental issues through top-down governance and control regulations. At a time when rural society in China is being transformed, we should recognize the influence of guanxi networks on the sharing of costs and benefits of control of pollution from pig farming, and which have some effect on rural environmental equalities. As rural society develops, along with improvements in institutional frameworks, guanxi networks should come to play a weaker role in rural environmental governance. In this situation, institutions will need to play a role in reducing the environmental inequalities induced by different guanxi networks.

In present study, we wanted to discover the changing of guanxi networks and the impact on the sharing of costs and benefits through a long period of observation, and the rural environmental inequality by pig farming pollution. The qualitative method enabled extensive insights into these issues—why the guanxi networks have changed, why the government treatment did not take effect, and why the farmers did organize and collectively do something.

However, the study has some limitations. First, the long-term observations at the village, in-depth interviews, and questionnaire surveys in this study were only conducted in S village, and thus the transferability of the results to other areas may be limited due to complex guanxi networks. Second, the study only considered the guanxi network of pig farmers and farmers, and other guanxi networks were ignored. Third, due to environmental cost, depreciation costs of pig houses are difficult to measure precisely, and we did not pay much attention to the precise measurement of cost benefits. Additional research is necessary and should use quantitative or experimental research methods to assess the cost and benefit of rural environment pollution governance through the perspective of guanxi networks.

## 6. Conclusions

In the quick transformation period in rural China, pollution from livestock and poultry breeding is not a simple environmental or economic problem. Guanxi plays an important role in the rural environmental pollution governance. Pig farming shaped the nature of guanxi in different groups. For the pig farmers, their guanxi networks are growing, while the farmers’ guanxi networks are shrinking. The different guanxi networks have an impact on the effectiveness of rural environmental pollution governance, and affect the way in which the costs and benefits of pollution control are shared; the rich have become richer and the poor have become poorer, and environmental inequalities have worsened. At a time when rural society in China is being transformed, we should recognize the influence of guanxi networks on the sharing of costs and benefits of controlling pollution related to pig farming, which and have some effect on rural environmental inequalities.

## Figures and Tables

**Table 1:** The scale of each group.

Item	Sample Class	Farmers		Pig farmers	
		Percentage (%)	Mean Value	Percentage (%)	Mean Value
Family scale	Not less than 4	42.2	5.3	45.7	5.2
	5 to 8	51.5		47.8	
	Over 8	6.3		6.5	
Educational level	Primary school and lower	68.8	/	67.4	/
	Junior middle school and above	31.2		32.6	
	Junior college and above	0.0		0.0	
Gender	Man	62.5	/	73.9	/
	Woman	37.5		26.1	
Age	Not less than 40	20.3	51.2	21.7	48.4
	41 to 50	34.4		41.3	
	Over 50	45.3		40.0	

**Table 2:** The capability of each group.

Item	Sample Class	Farmers		Pig Farmers	
		Percentage (%)	Mean Value	Percentage (%)	Mean Value
Gross income	Not Less than 10,000	12.5	54,840.6	7.6	76,349.1
	10,001 to 50,000	64.1		59.1	
	50,001 to 100,000	20.3		24.1	
	Over 100,000	3.1		8.2	
Pig breeding by relatives	None	45.3	1.6	30.4	1.9
	1 to 5	46.9		59.1	
	Over 5	7.8		10.5	
Village cadres or not	No	87.5	/	82.6	/
	Were	10.9		4.3	
	The proportion of Christianity	1.6		13.1	

**Table 3:** Costs and benefits of pig farmers.

Costs/Benefits	Contents	Data Standard	Remarks
Breeding cost	Feed cost	The cost of young pigs 200 RMB, the cost of medium pig 500 RMB, pig rearing large cost 1000 RMB.	The average weight of a piglet is 15–30 kg, a medium pig is 50–60 kg, and a large pig is 100–130 kg.
	Labor cost	one labor wage is 1500–2000 RMB/month; 8 pigs or more require 1 laborer to be responsible for feeding and management.	/
Environmental cost	Cost of treatment facilities (biogas digesters etc.)	The average cost of a biogas digester is 900 RMB/square meter; it costs about 10,000 to 20,000 RMB to build a biogas digester.	Only talk about the construction cost, ignore the manual processing and post-maintenance.
	External cost	Loss of neighborhood relations in the community.	/
Other costs	Depreciation cost of pig house	The cost of building a pig house is 70–90 RMB/square meter, and it will run for about 10 years.	The land belongs to the individual contracted land of pig farmers.
	Other	Disease prevention is 150 RMB, charges for water and electricity are 20 RMB, and loss due to pigs that died of disease is the equivalent of 30 RMB.	/
Benefits	Peak season	The price of a pig is 14–16 RMB/kg, a small part is 18–20 RMB/kg.	/
	Off-season	The price of a pig is 8–12 RMB/kg.	/
Government subsidies	Environment subsidies	The government will provide a subsidy of 1200 RMB when the biogas digester is checked and accepted.	/

## Data Availability

Data sharing is not applicable.
